# Supplementary material for: Are carbon emissions trading and green financial instruments synergistic? -Comprehensive quantitative research based on content analysis
Source: PLoS One. 2024 Mar 7;19(3):e0298601. doi: 10.1371/journal.pone.0298601 (PMC10919601; doi:10.1371/journal.pone.0298601)
Supplement: S1 Data — (ZIP) [file pone.0298601.s001.zip › Data/地级市Z_F工作报告.zip!/┴╔─■/╕╖╨┬╩╨/└┤╫╘╣1⁄2╓┌║┼í╛┬φ┐╦╩2╛▌═°í┐_2013.docx]

各位代表：

　　 现在，我代表市政府向大会报告工作，请予审议，并请市政协各位委员和其他列席同志提出意见。

　　一、过去五年工作回顾

　　过去五年，在市委的正确领导下，全市各族人民团结一心，奋力拼搏，超额完成了十四届人大一次会议确定的目标任务，开创了转型振兴的新局面。

　　——综合经济实力明显增强。主要经济指标增速高于全省平均水平，总量实现翻番。地区生产总值由2007 年的195.5 亿元增加到560 亿元，年均增长14.6%，其中一二三产业增加值年均增长10%、17.9%、13.7%；人均生产总值突破3万元；公共财政预算收入由11亿元增加到64 亿元，年均增长35.3%；累计固定资产投资1594 亿元，是上五年的4.1 倍；社会消费品零售总额由87.6亿元增加到203.4 亿元，年均增长18.4%。

　　——产业结构得到优化。三次产业比重调整为23：45：32，工业占比提高5个百分点，主导作用明显增强。大唐煤制天然气、巴新铁路等一批重大项目开工建设，液压、农产品加工、新能源等一批产业快速发展，多元化产业格局基本形成。规模以上工业企业达到570户，销售收入亿元以上企业达到110户，阜矿集团实现总收入240亿元。服务业发展水平提升，金融业、房地产业、旅游业加快发展。农业综合生产能力增强，畜牧业规模化养殖水平大幅提高，花生种植面积跃居全国第三位，粮食产量连续三年创历史新高。

　　——城乡基础设施和生态环境显著改善。完成了新一轮城市总体规划修编及一批专业规划编制工作。新城建设全面展开，“玉龙新城”核心区初具雏形，城市建成区面积由66平方公里增加到76.5平方公里。修建城市道路143条，主城区“六横六纵一环”道路框架基本形成；新增高速公路3条、210公里，路网密度跃居全省前列；新增地方普通公路60条、243公里；建成了日供水15万吨的引白水源工程、日处理16.5万吨的污水处理厂等一批重大基础设施。森林覆被率达到32.3%，城市环境空气质量二级以上天数由306天增加到347天。城镇化率达到56.6%，超过全国平均水平。

　　——人民生活水平普遍提高。城镇登记失业率控制在4.0%以内。1万平方米以上集中连片棚户区全部改造完成，改造面积330万平方米，惠及居民7.5万户。完成老旧小区环境综合整治619万平方米。社会保障覆盖面进一步扩大，社会保障及社会救助标准大幅提高。城镇居民人均可支配收入由8702元增加到17240元，年均增长14.7%；农村居民人均纯收入由4128 元增加到8850元，超过全国平均水平，年均增长16.1%。

　　——社会事业全面进步。新增省级以上工程技术研发中心20家，科技进步对经济增长贡献率由47% 提高到52%。“双高普九”通过省终

结验收，城区中小学全部铺设塑胶操场，改造农村校舍31.7 万平方米。建成了市图书馆等一批重点工程，农村文化站、社区文化广场等文化设施基本实现全覆盖。医疗卫生体制改革扎实推进，阜矿总医院外科楼、市中心医院病房楼、第二人民医院（妇产医院）等投入使用，医疗卫生机构每千人床位数由3.6张提高到4.2张。群众体育蓬勃开展，我市被确定为东北三省唯一的“全民健身示范城市”试点市。

　　 各位代表，刚刚过去的2012年，在宏观经济形势严峻复杂的情况下，我们牢牢把握“稳中求快、又快又好”的总基调，务实拼搏，奋力攻坚，保持了全市快速发展态势，主要经济指标增幅继续位居全省前列。

　　（一）加快产业集群建设，工业整体实力进一步增强调整和完善了“十大产业集群”规划布局和工作机制，产业集群发展思路、目标进一步明确，重大项目建设成效明显，产业规模迅速扩大。大唐煤制天然气一期项目完成投资180亿元。液压产业实现销售收入300亿元。氟化工、皮革基地晋升为省级开发区，与上海有机所、航天集团、徐工集团等成功合作，浙大液压创新中心投入使用，产业集群发展水平明显提升。

　　全年实施投资千万元以上项目超过1000个，其中新开工亿元以上项目150个。规模以上工业实现增加值210亿元，增长12.7%。民营经济增加值增长33.5%。高新技术产业增加值增长52%。

　　（二）加大城乡建设力度，城乡环境明显改善“玉龙新城”起步区路网基本形成，玉龙湖水系、绿化景观初具形象，市民文化中心、服务中心、绿地· 剑桥等项目加快建设，中泰世纪广场等一批项目落地。“沈彰新城”、“温泉新城”各有一批项目开工建设。

　　房地产施工面积超过1000万平方米。阜、彰两县被纳入省县城建设试点县。实现建筑业产值205亿元，增长26%。“五化工程”成效明显，新植树木588万株、栽摆鲜花840万株，铺设人行步道30万平方米，改造升级23条街路路灯，城市环境卫生状况有了较大改善。基础设施建设得到加强，阜盘高速、城市大外环、皮革产业大道建设加快推进，巴新铁路开始铺轨。改造城市道路43条，完成105个自然屯、309公里通油路工程。军民合用机场纳入国家民航“十二五”规划。细河综合整治二期工程全面实施，新增水面35万平方米。垃圾处理厂二期工程投入使用。全市造林129万亩。以凌河流域为重点的19个水污染治理项目开工。成功争创国家资源综合利用“双百工程”示范基地。章古台自然保护区晋升为国家级自然保护区。

（三）推进农业产业化发展，综合生产能力增强农产品加工重大项目建设走在全省前列，辉山、雨润等8个投资10 亿元以上项目在建，伊利乳业项目开始生产。农业结构调整步伐加快，花生面积达到280万亩；新建规模养殖小区339个，畜牧产业化水平居全省前列。粮食产量55亿斤。

　　农业生产条件得到改善，完成节水滴灌工程38.2万亩，农田综合机械化水平达到79.2%。建成国家级高产示范区30个。阜、彰两县分别被列为国家和省土地整理示范县。县域综合实力进一步提升，阜、彰两县公共财政预算收入分别增长32.1%、25.8%。

（四）突出集聚区建设，服务业发展水平提升中央商务区营业收入超过100 亿元，“温泉新城”晋升为省级现代服务业集聚区。大润发超市、鸿达购物广场等投入运营，兴隆CBD等项目建设进展顺利，宝地城等城市综合体项目开工。五金建材城等一批专业市场建设加快。旅游业稳步发展，温泉开发、乡村游加快兴起。金融部门加大支持经济发展力度，新增贷款95亿元。交通银行阜新分行获批筹建。

　　（五）扩大对外开放，招商引资成效显著招商引资到位额405 亿元，增长34.1%；直接利用外资1.8 亿美元，增长22%；出口总额1.8亿美元，增长17.6%。

　　先后组织7 次大型招商活动，签约额263 亿元。香港盈德煤化工、大连远东铜业等一批重大工业项目和国际玛瑙城、农产品物流城等一批重大服务业项目签约落地。推动企业联合重组，五粮液集团重组三沟酒业项目积极推进，101 户企业实施合资合作。成功举办了液压装备制造业展览会、玛瑙博览会和氟化工产业高峰论坛，扩大了阜新对外影响力。

　　（六）着力保障和改善民生，人民生活质量持续提高省、市确定的45件惠民实事全部完成。新增实名制就业6.4 万人次。退休人员基本养老金月人均增加182元。城镇居民基本医疗保险补助标准提高到240元，参保率达到97.2%，基本实现市级统筹及“一卡通”结算。医疗保险门诊统筹报销比例提高20%，门槛费降低60%。城乡低保年补助标准分别提高到3960 元和2010 元。为70%以上的城乡居民建立规范化电子健康档案。建立社区职工帮扶站182个，实行工资集体协商企业110户。继续推进国有工矿和林区、垦区棚户区改造，1.25万户低收入家庭得到安居保障。实施居民楼节能改造121万平方米。住房公积金在全省率先实现城乡全覆盖。

　　市人民公园、博物馆免费开放，市老年活动中心主体完工。建设村文化广场276个。解决80个自然屯广播电视盲点问题。新建农村社区服务中心60个、农村学校塑胶操场23块。农村饮水安全工程使8.5万人受益。“慈善建房”救助项目荣获第七届中华慈善奖。13.8万农村低收入人口年人均纯收入达到2200 元以上，争取“国家扶贫改革试验区”工作取得积极进展。

（七）全面发展社会事业，社会管理创新得到加强新建标准化学校44所，农村6 所初中进县城，新建改扩建幼儿园19 所。市实验中学实现跨地区招生。市二职专新校区建成。公共文化服务体系不断完善，文化事业和文化产业加快发展。辽宁工大在人才培养、科技服务等方面为阜新作出重要贡献。

　　城市社区卫生服务体系进一步完善，农村三级医疗卫生服务网络基本形成。顺利通过省级卫生城复检。幸福家庭促进计划全面推进，计划生育服务水平提升。首届全民健身运动大会取得圆满成功，“十二运”比赛场馆改造完成。

　　社会矛盾大排查大调处活动深入开展，一批信访问题得到妥善化解。社会治安防控体系不断强化，治安状况持续好转。安全生产形势总体平稳。双拥共建工作扎实推进。民族团结进步事业不断发展。加强政府自身建设，深入开展政德教育、“下基层、保增长”和“行政效能大提升”等活动，促进政府系统工作效率和服务水平提高。审计监督成效明显。坚持向人大及其常委会报告工作和政协通报重要情况制度，办复人大代表建议111件、政协提案148件。

　　全市各行各业、各条战线都呈现出新变化，统计、物价、法制、地震、人防、民防、食品药品监督、地方志、档案、妇女儿童、外事侨务、对台事务、残疾人、红十字会、宗教等工作取得新成绩，供电、工商、烟草、质监、气象、邮政、通讯、石油、武警、消防等单位为地方经济发展作出了重要贡献。

　　各位代表，过去的五年，是阜新综合实力提升最快、城乡面貌变化最大、社会建设成效最好、人民群众得到实惠最多的五年。五年来，我们抓住了加快发展的重要机遇，探索出了一条符合阜新实际的发展路子，构筑起了具有特色优势的产业框架，夯实了转型振兴的坚实基础。五年来，我们坚持以艰辛换发展，以实干创业绩，凝聚了自强不息、奋发向上的精神力量，推动全市经济社会发展进入了新时期。

　　这些成绩的取得，是省委、省政府高度重视、亲切关怀的结果，是市委总揽全局、正确领导的结果，是市人大和市政协大力支持、有力监督的结果，是各民主党派、工商联、无党派人士、各人民团体和社会各界人士广泛参与、群策群力的结果，是全市广大干部群众团结拼搏、艰苦奋斗的结果。在此，我代表市政府，向所有关心、支持和参与阜新建设的同志们、朋友们，表示崇高的敬意和衷心的感谢！

　　 同时，我们也清醒地认识到，政府工作中还存在一些矛盾和问题。主要是，经济总量小、综合实力弱；工业规模和产业水平有待提高，现代服务业发展不够快，农业基础设施建设还需加强；城乡居民收入仍处于全省较低水平；生态环境还很脆弱，节能减排压力较大；个别政府部门服务质量、行政效能有待提高。这些矛盾和问题必须在今后的工作中认真加以解决。

　　二、今后五年的奋斗目标

　　今后五年，是阜新实现转型振兴的重要时期，是全面建成小康社会的关键时期。我们将深入贯彻落实党的十八大精神，紧紧围绕转型振兴大局，以做大经济总量、提高发展质量、改善人民生活为目标，深化改革、扩大开放，着力推进产业集群化、新型城镇化、农业现代化进程，着力加强基础设施、生态环境、民生工程建设，促进产业优化升级、城乡环境生态宜居、社会事业全面进步，为早日全面建成小康社会奠定坚实基础。

　　今后五年全市经济社会发展的奋斗目标是：

　　——经济总量实现倍增。地区生产总值达到1200亿元，年均增长13%；人均GDP达到6万元；公共财政预算收入150 亿元，年均增长18.6%；累计固定资产投资5000 亿元，年均增长25%。发展速度高于全省平均水平，综合经济实力大幅提升，经济发展协调性进一步增强。

　　——产业结构不断优化。三次产业结构调整为12.5：50：37.5。重点产业集群和骨干企业进一步做大做强，工业占比提高5个百分点，主导作用进一步凸显。旅游、金融、房地产等现代服务业加快发展，服务业占比提高5.5个百分点。现代农业加快发展，综合生产能力明显增强。

　　——城乡面貌实现更大改观。城市总体规划布局更加科学，“玉龙新城”等新城核心区基本建成，老城区改造成效明显。实施机场、辽西北供水工程等一批重大基础设施项目，城市功能日趋完善。县城改造建设步伐加快，以中心镇为重点的小城镇建设全面推进。到2017年，城镇化率达到67%以上，实现城市建成区100平方公里、100万人口目标。

　　——改革开放取得新突破。深化行政管理、财政体制、投融资体制等重点领域、关键环节改革，体制机制创新取得明显成效。沈阳经济区一体化建设、市矿校合作进一步深化，对外开放水平明显提高。引进内资、外资和出口总额年均增长20%以上。

　　——生态环境持续改善。深入推进生态文明建设，推行绿色发展、循环发展、低碳发展，矿产、土地等资源开发利用效率进一步提高，水、大气、矿山等环境治理取得新进展，节能减排任务全面完成。到2017 年，森林覆被率达到40%以上，城市环境空气质量二级以上天数保持在

340天以上。

　　——民生工程成果显著。坚持为民办实事、解难事，推进民生工作具体化、职能化、制度化。就业渠道和岗位明显增多，覆盖城乡的社会保障和救助体系不断完善，城乡居民富裕程度普遍提高，努力让人民拥有更稳定的工作、更满意的收入、更可靠的保障、更舒适的环境。到2017年，城镇居民人均可支配收入35000 元，农村居民人均纯收入17500元，均实现翻番以上增长。——社会事业全面发展。各级各类教育均衡发展，教育教学质量明显提高；科技进步对经济增长贡献率达到60%；文化事业和文化产业协调发展；医疗卫生体制改革取得成效；群众体育和竞技体育全面发展。社会管理创新水平提升，社会和谐稳定。

　　三、2013 年工作安排

　　按照市委十一届三次全会总体部署，今年政府工作的总体要求是：全面贯彻落实党的十八大精神，坚持以中国特色社会主义理论体系为指导，牢牢把握“稳中求进、又好又快”的总基调，以科学发展为主题，以转变经济发展方式为主线，以提高经济增长质量和效益为中心，以改革开放为动力，以项目建设为核心，以推进工业化、城镇化为重点，加快经济发展，构建和谐阜新，为实现转型振兴而努力奋斗。主要预期目标是：地区生产总值完成660亿元，增长13%；公共财政预算收入76.3亿元，增长19.1%；固定资产投资630亿元，增长26%；城镇居民人均可支配收入19500 元，增长13.1%；农村居民人均纯收入10000元，增长13%。

　　（一）加快项目建设步伐，着力扩大投资规模发挥投资拉动经济增长的主动力作用，坚持以项目为核心，优化投资结构，提高投资质量和效益，以投资做大总量、做强财力、调整结构。开展“项目建设年”活动，全年续建投资千万元以上项目600个；新开工投资5000万元以上项目300 个。

　　突出抓好重大项目建设，新开工投资亿元以上项目150 个。其中，10亿元以上项目20个，5—10亿元项目30个，1—5亿元项目100个；工业项目70个，服务业项目20个，农业项目10个，城市建设及房地产项目35个，基础设施项目11个，社会事业项目4个。

　　拓宽项目渠道，提高项目质量。把握国家、省投资方向和发达地区产业转移趋势，围绕我市产业发展定位，加大项目储备力度，研发包装推介一批重大项目。

　　完善工作机制，提升项目工作水平。全力抓好项目规划、招商、落地、开工、投产、达产等关键环节，促进项目早开工、早投产、早达产。

　　（二）突出工业主导地位，加快产业集群建设全面推进工业“五项工程”，促进工业总量做大、效益提升。规模以上工业实现增加值260亿元，增长15%。

　　推动“十大产业集群”上规模、上水平。加快煤化工基地核心区基础设施建设，大唐煤制天然气第一系列项目竣工投产，开工建设北京国能煤焦油和粗酚加工、大唐LNG 等项目，加快大唐煤制天然气第二、三系列、香港盈德煤化工等项目前期工作。液压产业实现销售收入400亿元，农产品加工业实现250亿元，氟化工、皮革、铸造等产业超过100亿元，板材家居、新型材料、玛瑙等产业实现30%以上增长。开工建设德尔汽车转向泵扩产升级、联化科技二期、韩国长川制鞋、保利民爆二期等项目，推动航天神坤高端液压支架、万联铸件、彰武雨润等项目竣工投产。积极推进富国集团等企业整体搬迁、日本新型建材园等项目。加强与上海有机所、航天集团、徐工集团合作，促进产业升级。积极创建国家新能源示范市。

　　做大做强现有企业。支持阜矿集团、发电公司等企业发展，扶持40 户企业尽快进入10 亿元企业行列。新增规模以上企业80户，新增销售收入超亿元企业20户、10亿元企业5户，实现倍增企业100户。实施技改项目260个，完成投资180亿元。支持中小企业特别是小微企业发展，民营经济增加值实现30%增长。

　　加强科技创新，推进氟化工研发中心、天士力中药研发基地等竣工。推进工业化、信息化深度融合，打造企业网上交易平台。做好节能减排和淘汰落后产能工作。

（三）加大招商引资力度，提高对外开放水平推动对外开放扩大领域、提升层次，全方位引进项目、资金、技术、人才。招商引资到位额500亿元，增长23.5%；直接利用外资2 亿美元，增长9.3%；出口总额2亿美元，增长11.1%。

　　围绕工业产业集群、服务业集聚区、新城新市镇等重点领域，突出珠三角、长三角、港澳台等重点地区，大力开展主题概念招商、专业招商。强化企业招商主体作用，促进以商招商。全年签约落地投资10 亿元以上项目50个。深化区域合作。做好沈阳经济区综合配套改革相关工作，加快沈阜一体化进程。

（四）以集聚区建设为重点，加快发展现代服务业以服务业增速高于全市经济增速为目标，推进服务业总量做大、水平提升。完成社会消费品零售总额235亿元，增长15.5%。加快服务业集聚区建设。扩大中央商务区、站前服务业集聚区、“温泉新城”等服务业集聚区规模，加快“玉龙新城”CBD建设，推进兴隆CBD、六合国际城市综合体、站前现代城等项目竣工，开工建设红星美凯龙商业综合体、中泰世纪广场等项目。大力发展物流业。世通物流园一期投入使用，开工建设农产品物流城、香港豪德物流园、海州煤炭物流园等项目。培育发展电子商务、连锁经营等新兴业态。推进“无线城市”建设，提高城市信息化水平。发展旅游产业。推进海棠山、瑞应寺、大清沟等重点景区开发建设。完善乡村旅游产业体系，积极发展沟域旅游经济。加快发展金融业。实现直接融资100亿元，间接融资100亿元。推动交通银行阜新分行开业，积极引进域外金融机构。充分发挥证券、信托、租赁等融资渠道作用，扩大社会融资总规模。支持保险业加快发展。继续实施“三年倍增”计划，以工业产业集群和服务业集聚区建设为重点，加快城区经济发展。

（五）大力发展现代农业，促进农民稳定增收全面落实强农惠农富农政策，促进农民增收、农业增效、农村发展。

　　积极发展现代农业。推进农业产业化经营，引进培育壮大一批龙头企业。加快推进农业结构调整，抓好“一县一业”，稳定花生种植面积，提高深加工能力。大力发展特色高效农业，积极培育发展新奇特、名贵优产品。新建改造规模养殖小区300个。稳定粮食生产，提高单产水平。

　　改善农业基础条件。实施节水滴灌工程40万亩。新建各类饮水工程100处。农田综合机械化水平达到82%。加强农村社会事业建设，促进城乡基本公共服务均等化，改善农村生产生活条件。加强村屯规划，整治村容村貌。

　　拓宽农民增收渠道。引导和鼓励农民从事非农生产和经营，积极培养联户经营、专业大户、家庭农场等新型经营主体，稳妥推进农村土地承包经营权流转。

　　以工业集群化、农业产业化为重点，加快发展县域经济，提高县域发展实力。

　　（六）加快城镇化步伐，打造生态宜居城市

　　坚持以城镇化建设为新动力，以生态宜居为目标，促进城市扩容提质，城镇化率提高2个百分点。

　　加快新城建设。全面完成“玉龙新城”核心区配套设施建设，提升景观建设水平，“两馆一心”等项目投入使用，市民服务中心主体完工，开工建设恩典国际商住开发等37 个项目。完善“沈彰新城”基础设施，开工建设绿色智慧谷等20个项目。加快“温泉新城”配套设施及信达·泉天下等项目建设。推进老城改造，全年拆迁90万平方米，开工建设320 万平方米。抓好7个城中村、3个城边村改造，加快城区全域城市化进程。

　　完善县城改造建设规划，阜彰两县新增开发面积均超过100万平方米。加快小城镇建设，重点打造以产业为支撑的特色小城镇。

　　坚持优化结构、提升档次、有序开发的原则，促进工业、住宅、商业、旅游、文化地产等多元发展。房地产完成投资100亿元。实现建筑业总产值240亿元，增长14.3%。

　　加强基础设施建设。阜盘高速及北延伸线、城市大外环、沈彰开发大道、皮革产业大道等项目竣工，巴新铁路阜新至赤峰段全线通车。配合做好京沈客专、辽西北供水、锦阜高铁路扩能改造等工程实施。力争军民合用机场项目核准并开工。改造拓宽城市道路32条。新建通屯油路150公里。全面完成细河综合治理二期、玉龙湖水系二期工程。继续抓好供热、供水、供气等地下管网改造。

　　继续实施“五化工程”，提高绿化水平、硬化质量、亮化档次、净化标准、美化品位，努力让城市面貌焕然一新。

　　加快推进青山工程、碧水工程、蓝天工程建设。全年植树造林100 万亩，200万亩经济林工程提前一年完成；实施矿山生态治理1.1万亩。开发区、“沈彰新城”等污水处理厂投入使用，开工建设清河门污水处理厂二期和旧庙镇、大巴镇污水处理厂及养息牧河人工湿地。依法科学管理国土资源，推进资源优化利用。提升城市管理水平。集中整治城市交通、户外经营等秩序，加强城市基础设施、公共服务设施等管理，积极创建国家级卫生城。

　　 （七）坚持民生为先，让发展成果更多地惠及百姓

　　把保障和改善民生放在更加突出的位置，公共财力优先保障民生，重点实施十项民生工程。

　　1．就业工程。新增实名制就业5万人，扶持创业带头人330人，开展创业培训2300人，职业技能培训1.5万人。

　　2．社会保障工程。继续扩大社会保障覆盖面，城乡低保、农村五保补助标准提高10%，重点优抚对象救助保障和抚恤标准提高15%。开展企业和谐劳动关系建设，推进民营企业工资集体协商。

　　3．安居工程。为1.4万户低收入家庭提供住房保障。完成住宅楼节能改造100万平方米。新增区域一体集中供热面积300万平方米、煤气用户8000 户。做好住房公积金归集及使用工作。

　　4．教育基础工程。建设标准化学校50所，新建改扩建幼儿园12 所，改造维修校舍5万平方米。

　　5．文化惠民工程。新建市县数字图书馆，新增社区书屋46个、职工书屋100个。

　　6．健康工程。开工建设第二人民医院儿科综合楼、蒙医药研究所综合楼。社区卫生服务中心合格率达到80%。城乡居民规范化电子健康档案建档率达到75%以上。

　　7．农村环境工程。解决6 万人饮水安全问题。建造无害化厕所1万座。

　　8．便民工程。新增6 条公交线路。建设农超对接蔬菜连锁经营店20家。

　　9．扶贫工程。启动“国家扶贫改革试验区”建设，10万低收入人口年人均纯收入超过2500元。为100 户农村特困户新建住房。充分发挥慈善和红十字会作用。

　　10．敬老工程。阜彰两县分别建成1万平方米的农村困难家庭常年病人托管中心，实施市福利中心改扩建工程。

　　（八）加强社会建设，进一步推进社会管理创新

　　办好人民满意教育，促进义务教育均衡发展。继续做好以提高教育教学质量为目标的教学改革。提高职业教育对产业和社会服务能力。鼓励和支持民办学历教育和技能教育。做好民族教育和特殊教育。

　　大力发展公益文化事业，组织好群众喜闻乐见的文化活动，积极筹备参加省第九届艺术节。开工建设市传媒大厦。推进文化事业单位改革。

　　深化医药卫生体制改革，巩固基本药物制度，完善公共卫生和医疗服务体系。基本建成县区、乡镇、村组食品安全工作网络体系。实施幸福家庭促进计划，提高出生人口素质。

　　承办好“十二运”分会场赛事，大力推进全民健身场地设施建设，争创国家“全民健身示范城市”，促进群众体育和竞技体育全面发展。

　　加强和创新社会管理。强化社会治安综合治理工作，依法防范和惩治违法犯罪活动。强化公共安全体系和企业安全生产基础建设，积极推进消防安全网格化管理，遏制重特大事故发生。落实领导干部信访责任制、矛盾纠纷排查调处机制，有效化解积案、预防新案。积极开展双拥共建活动。积极培育民族特色文化和产业，大力支持民族地区加快发展。全面落实党的宗教政策，促进宗教和谐稳定。做好“六五”普法工作，完善法律援助体系。组织好第三次全国经济普查工作。

　　加强民主法治建设。主动接受人大的法律监督和政协的民主监督，认真办理人大代表的议案、建议和政协提案、委员意见，充分听取各民主党派、工商联、无党派人士及各人民团体的意见，保障和支持司法机关依法履行职责，广泛接受社会公众和新闻舆论监督。做好村民委员会换届选举工作。

（九）加强政府自身建设，努力打造人民满意的政府

　　全面建成小康社会是全市人民共同期盼的目标，新一届政府肩负着重要使命。我们必须以改革创新的精神，抓好政府自身建设，努力建设对人民负责、为人民造福、令人民满意的政府。

　　坚持解放思想、开拓创新。组织政府系统深入学习贯彻党的十八大精神，把广大干部的思想和行动统一到全面建成小康社会的目标任务上来。坚持以改革的精神、创新的理念谋划发展、惠及民生，用新思路、新办法破解难题、推进工作。深化行政审批制度改革，适时取消和调整一批行政审批项目。坚持科学决策、民主决策，广泛听取社会各界意见和建议，使决策更加科学合理。切实转变作风、真抓实干。坚持重心下移、调查研究，深入基层、深入一线，了解真实情况，研究解决实际问题。坚持实干富民、实干兴市，多做打基础、利长久、增后劲的工作。对于确定的事项，都要制定方案、路线图、时间表，层层抓好落实。精简会议文件，坚持开短会、讲短话、发短文，减少事务性工作。完善政府绩效考核体系，加强对重点工作、重点项目的督促检查，确保政令畅通。严格控制机构编制。

　　积极践行宗旨、造福于民。坚持深入群众、了解民意，带着深厚感情、强烈责任做好群众工作。进一步畅通民众诉求渠道，切实维护群众合法权益，多为群众做一些看得见、摸得着的实事。深入推行政务公开，加快电子政务平台建设，及时公开群众普遍关心的政府信息。推进乡镇、街道便民服务中心建设。完善政府诚信体系，坚持少说多做，坚决兑现承诺，提高政府公信力和执行力。

　　坚持以身作则、勤政廉政。不断完善惩治和预防腐败体系，继续推进“五大系统”建设。加强廉洁自律教育，筑牢思想防线。加强重大资金、重大项目监察监管，进一步加大领导干部经济责任审计力度。坚持依法行政，着力整治庸懒散奢等不良风气，严肃查处不作为、滥作为行为。认真落实党风廉政建设责任制，严肃查处违法违纪行为，树立干部清正、政府清廉、政风清明的良好形象。

　　各位代表，阜新已经进入新的发展时期。我们坚信，有市委的坚强领导，有全市各族人民的奋力拼搏，阜新转型振兴的宏伟目标一定能够实现，阜新人民的生活一定会更幸福、更美好！
